# Sulforaphane inhibits self-renewal of lung cancer stem cells through the modulation of sonic Hedgehog signaling pathway and polyhomeotic homolog 3

**DOI:** 10.1186/s13568-021-01281-x

**Published:** 2021-08-23

**Authors:** Fanping Wang, Yanwei Sun, Xiaoyu Huang, Caijuan Qiao, Wenrui Zhang, Peijun Liu, Mingyong Wang

**Affiliations:** 1 Henan Key Laboratory of Immunology and Targeted Drug, School of Laboratory Medicine, Xinxiang Medical University, Xinxiang, 453003 Henan China; 2 Key Laboratory for Tumor Precision Medicine of Shaanxi Province, First Affiliated Hospital of Xi’an Jiaotong University, Xi’an, 710061 Shaanxi China; 3 Xinxiang Key Laboratory of Immunoregulation and Molecular Diagnostics, Xinxiang, 453003 China

**Keywords:** Sulforaphane, Lung cancer, Cancer stem cell, Sonic Hedgehog Signaling Pathway, Human polyhomeotic homolog 3

## Abstract

Sulforaphane (SFN), an active compound in cruciferous vegetables, has been characterized by its antiproliferative capacity. We investigated the role and molecular mechanism through which SFN regulates proliferation and self-renewal of lung cancer stem cells. CD133^+^ cells were isolated with MACs from lung cancer A549 and H460 cells. In this study, we found that SFN inhibited the proliferation of lung cancer cells and self-renewal of lung cancer stem cells simultaneously. Meanwhile, the mRNA and protein expressions of Shh, Smo, Gli1 and PHC3 were highly activated in CD133^+^ lung cancer cells. Compared with siRNA-control group, Knock-down of Shh inhibited proliferation of CD133^+^ lung cancer cells, and decreased the protein expression of PHC3 in CD133^+^ lung cancer cells. Knock-down of PHC3 also affected the proliferation and decreased the Shh expression level in CD133^+^ lung cancer cells. In addition, SFN inhibited the activities of Shh, Smo, Gli1 and PHC3 in CD133^+^ lung cancer cells. Furthermore, the inhibitory effect of SFN on the proliferation of siRNA-Shh and siRNA-PHC3 cells was weaker than that on the proliferation of siRNA-control cells. Sonic Hedgehog signaling pathway might undergo a cross-talk with PHC3 in self-renewal of lung cancer stem cells. SFN might be an effective new drug which could inhibit self-renewal of lung cancer stem cells through the modulation of Sonic Hedgehog signaling pathways and PHC3. This study could provide a novel way to improve therapeutic efficacy for lung cancer stem cells.

## Introduction

Lung cancer is one of the most prevalent cancers which accounts for approximately one-fourth of the cancer incidence and is the second leading cause of death both in china and in developed countries around the world (Torre et al. 2016; Siegel et al. 2019). Despite initial treatment with conventional therapy has a high remission rate, eventually the disease almost always relapses in the form of resistance to chemotherapy and radiation therapy, therefore the overall 5-year survival rate of patients is less than 5% (Bray et al. 2018).

Increasing evidence of the existence of cancer stem cells (CSCs) in lung cancer explains why standard chemotherapy or radiotherapy regimens against lung cancer are usually ineffective and result in further tumor recurrence and progression (Hermann et al. 2010). CD133 is a cell membrane glycoprotein and it contains five transmembrane rings. It has been reported that CD133^+^ subpopulation of multipotent cells has the biological features of CSCs which possess extremely proliferative and self-renewal characteristics (Kim and Ryu 2017; Mizugaki et al. 2014). Although CSCs are only a small part of tumors, they have a powerful ability to self-renew and divide. The tumors rich in CSCs are more aggressive and lead to worse clinical outcomes. Therefore, the development of therapeutic strategies and drugs that specifically target CSCs can eradicate tumors effectively and reduce the risk of recurrence and metastasis.

Sulforaphane (SFN) is an isothiocyanate (ITC) found in cruciferous vegetables such as broccoli and cabbage (Clarke et al. 2008; Sangthong et al. 2016). A series of clinical trials have demonstrated that SFN can inhibit the malignant growth of cancer cells such as pancreatic cancer cells and breast cancer cells and has no obvious toxicity to the normal cells (Fisher et al. 2017; Jaman et al. 2018; Srivastava et al. 2011; Subramaniam et al. 2018). Therefore, it has been safely suggested using SFN as a potential candidate for cancer treatment. However, the effect of SFN on the development of lung cancer and its mechanism is still unknown.

Sonic Hedgehog (SHH) signaling pathway has an essential role in the control of stem cell growth in embryonic tissues, and it plays a key role in the development of tissues and organs (Varjosalo et al. 2008). Recent evidence suggests that the SHH signaling pathway contributes to tumorigenesis when it is mutated or misregulated (Subramani et al. 2017; Song et al. 2017). Since the SHH pathway plays a critical role in the renewal of CSCs, blockade of SHH pathway has evolved as a promising therapy for various types of cancers including lung cancer (Savani et al. 2012; Kieran 2014). Sonic hedgehog (Shh), Smoothened (Smo) and Gli1 are important factors in the SHH signaling pathway.

Polycomb group molecule human Polyhomeotic Homolog 3 (PHC3) is a member of the PcG protein family (Robinson et al. 2012), and PcG is a family of chromatin-related gene silencing proteins that regulates gene expression program in epigenetics. PcG proteins have essential roles in early embryonic development and have been implicated in embryonic stem cell pluripotency (Boyer et al. 2006). The PRCs silence tumor-suppressor genes by histone modifications, leading to cancer cell proliferation, metastasis and drug resistance (Crea et al. 2013). Thus, PHC3 similar to SHH signaling pathway, plays an important role in controlling the growth of stem cells in early embryonic development. However, what role PHC3 and SHH signaling pathways play in human lung CSCs still needs to be elucidated.

This study is based on the hypothesis that SFN inhibits self-renewal of lung CSCs by regulating SHH signaling pathway and PHC3. In the present study, SFN can inhibit the proliferation of lung cancer cells and self-renewal of lung CSCs. And SHH signaling pathway and PHC3 were activated in lung CSCs. Importantly, the results revealed that SFN is a potent drug preventing lung cancer cell growth through regulating SHH signaling pathway and polycomb group molecule PHC3.

## Materials and methods

### Cell culture

Human non-small cell carcinoma of the lung cancer A549 and H460 cell lines were obtained from the Cancer Institute of Southern Medical University (Guangzhou, China). The cells were maintained in a humidified atmosphere of 5% CO_2_ at 37 °C in DMEM culture medium (Hyclone) with 10% fetal bovine serum (Gibco). The lung CSCs which express CD133 were isolated by the BD MACs and identified by flow cytometry. CD133-positive cells were cultured in MEBM basal medium (CBM, New Jersey, USA) to maintain the characteristics of stem cells.

### Cell viability assay

Cells in logarithmic phase were seeded into 96-well plates at a cell density of 1 × 10^4^/well, and then different concentrations of SFN were added into each well and incubated together for 48 h. The final concentrations of SFN were 0, 2, 4, 6, 8, 10, 12 μmol/L, respectively. Then, 10 μL MTT reagent of concentration 5 μg/L was added into the cell medium of each well and incubated for 4 h at 37 °C. Following the removal of the supernatant, 150µL dimethyl sulfoxide (DMSO) was added to dissolve formazan. The absorbance at 490 nm was measured with a microplate reader (Beckman Coulter, Brea, CA, USA). Each reaction was performed in triplicate. At the same time, changes in cell density were observed by optical microscope.

### Isolation and identification of lung cancer stem cells

CD133^+^ cells were obtained from A549 and H460 cells using CD133 Microbeads by MiniMACS separator (MiltenyiBiotec, Bergish Gladbach, Germany). A549 and H460 cells were collected separately by centrifugation. Different groups of cells (1 × 10^8^ cells/sample) were resuspended in 500 μl of the degassed buffer, respectively. Then the cell suspension was added onto the prepared column. The unlabeled cells (CD133^−^) were collected as they passed through the columns. MS columns were washed with degassed buffer. Then the column was removed from the separator and placed on a new suitable collection tube. Buffer was pipetted onto the column, and a fraction was immediately flushed out with the magnetically labeled cells (CD133^+^) by firmly applying the plunger supplied with the column.

### Flow cytometry analysis

Cells were collected separately by centrifugation, then the cells were washed twice with PBS solution, and up to 1 × 10^6^ cells were resuspended in 500 μl of PBS, respectively. The cells were then incubated with PE mouse anti-human CD133 for 30 min at room temperature in the dark. The positive cells were detected using a flow cytometer (BD Biosciences, San Jose, CA, USA) and were analyzed by FlowJo 9.1 software.

### TumorSphere formation assay

Cells were placed in 6-well ultralow attachment plates (Corning Inc.) at a density of 1,000 cells/mL in tumorsphere culture medium DMEM (Invitrogen, Carlsbad, CA, USA) supplemented with 1% N2 supplement, 2% B27 supplement, and 100 ng/mL epidermal growth factor at 37 °C in a humidified atmosphere of 95% air and 5% CO_2_. These cells were then treated with different concentrations of SFN at the same time. Primary spheroids were collected following 14 days of culture, and tumorspheres were measured using an inverted microscope system (magnification, Eclipse Ti‑s, Nikon, Tokyo, Japan).

### Reverse transcription and QPCR analysis

The total RNA was extracted from cells with Trizol reagents. cDNA was synthesized from 1 μg of mRNA with a high capacity cDNA reverse transcription kit according to the manufacturer’s instructions. Subsequently, cDNA was amplified by QPCR with the SYBR Premix Ex Taq kit according to the manufacturer's instructions using the ABI7300 Sequence Detection System. PCR conditions were as follows: one cycle at 95 °C for 3 min, followed by 40 cycles at 95 °C for 30 s and 64 °C for 1 min. All assays were performed in triplicate and were calculated on the basis of ∆∆Ct method. The n-fold change in mRNAs expression was determined according to the method of 2^−∆∆CT^. Te primers used for QPCR are listed in Table 1.

**Table 1:** List of primers used for QPCR

Gene	Forward (5’ → 3’)	Reverse (5’ → 3’)
Shh	CGCACCTGC TCTTTGTGG	GGAGCGGTTAGGGCTACTCT
Smo	TCGCTA CCCTGCTGTTATTC	GACGCAGGACAGAGTCTCAT
Gli1	CTG GATCGGATAGGTGGTCT	CAGAGGTTGGGAGGTAAGGA
PHC3	AGTGGGGAGAGGAGAAGA	GGTGGTGGAACAGAA ACA
β-actin	AGAGCTACGAGC TGCCTGAC	AGA GCT ACG AGC TGC CTG AC

### Western blotting

Protein sample was extracted from cells with an ice-cold SDS protein lysis buffer. Protein concentration was measured by a Micro BCA Protein Assay Reagent kit. Then protein sample was separated by 10% SDS-PAGE electrophoresis and transferred onto 0.45 mm PVDF membranes. The membranes were blocked with 5% non-fat milk in TBST buffer for 1 h, incubated overnight with primary antibody at 4 °C and then incubated with secondary antibody for 1 h at room temperature. The antigen–antibody complexes were visualized using the ECL detection system. The analysis of the bands was conducted by the Image J software. The following antibodies were purchased from commercial sources including anti-Shh (ab53281), anti-Smo (ab32575), anti-Gli1 (ab134906), anti-PHC3 (GTX32785) and anti-GAPDH (CWBIO).

### RNA interference

siRNAs for SHH and PHC3 were purchased from Invitrogen and the sequences are listed in Table 2. Cells were seeded in 6-well plates at a cell density of 3 × 10^5^ cells/well in 10% serum medium without antibiotics. After 24 h, cells were transfected in Opti-MEM using LipofectamineRNAiMAX (Invitrogen) and the 20 nmole siRNA was resuspended according to manufacturers instructions. The cells treated with Stealth negative control med component of kit (#12,935,300, Invitrogen) were served as controls (si-control). siRNA transfection efficiency in cells was assessed by Western-blotting.

**Table 2:** List of primers used for siRNA

si-RNA	S Sequence (5’ → 3’)	AS Sequence (5’ → 3’)
Shh	ACAGGCUGAUGACUCAGAGGUGUAA	UUACACCUCUGAGUCAUCAGCCUGU
PHC3	CAGAGUUGUUGCUAUACAAGG	UUGUAUAGCAACAACUCUGGA

### Statistical analysis

Statistical analysis was performed using SPSS16.0 software. Data presented were mean ± SD from three different experiments. Statistical significance between different groups was determined using Students *t*-test. A value of *P* < 0.05 was considered statistically significant.

## Results

### SFN inhibits the growth of lung cancer cells and self-renewal of lung CSCs

**Fig. 1:**
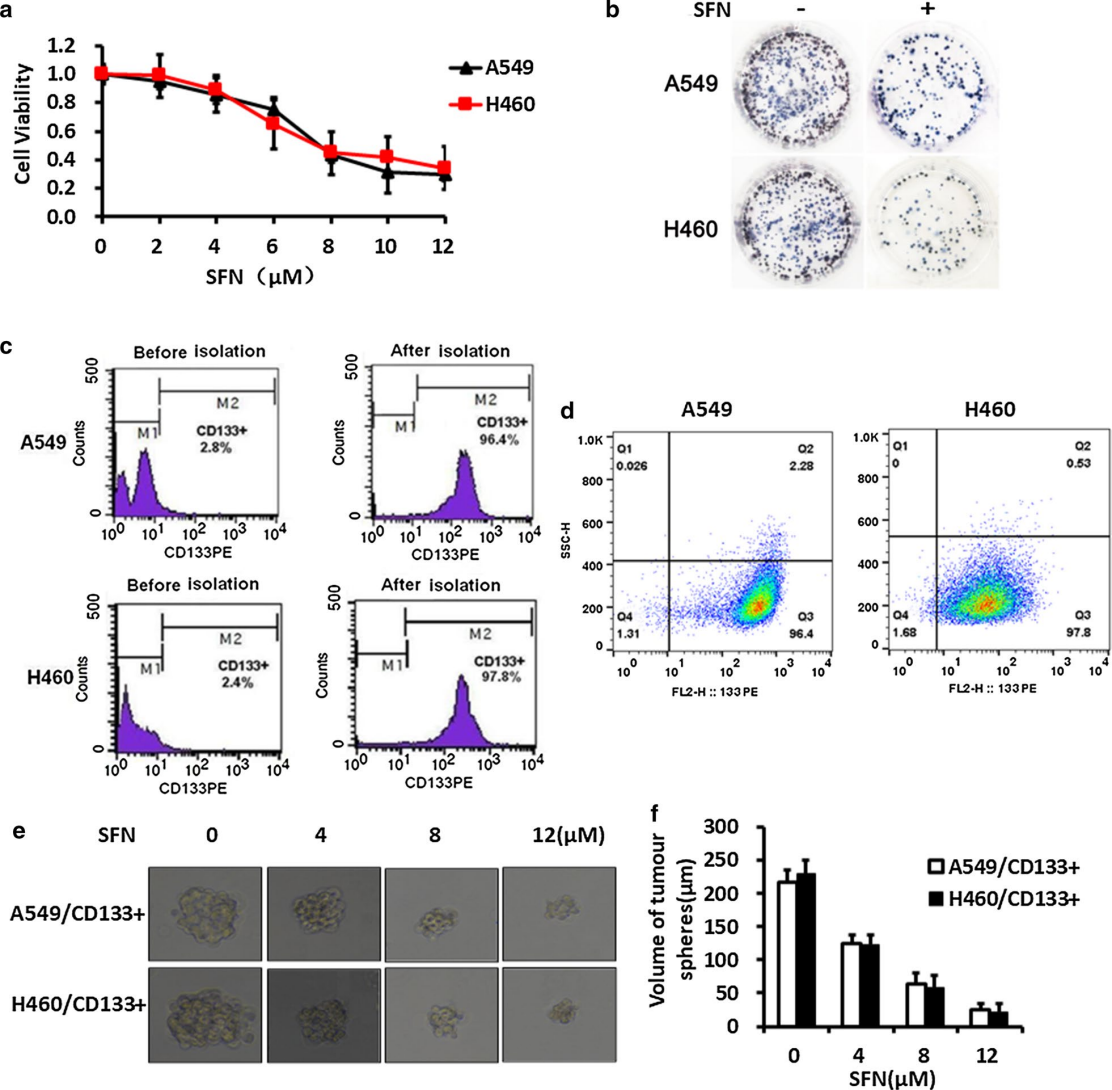
Inhibitory effect of SFN on cell proliferation. **a** Inhibitory effect of SFN on viability of A549 and H460 cells in a dose-dependent manner following treatments with 0, 2, 4, 6, 8, 10 and 12 μmol/L SFN for 48 h by MTT assay. **b** Inhibitory effect of SFN on cell density of A549 and H460 cells following treatments with 8 μmol/L SFN for 48 h by optical microscope. **c**, **d** CD133^+^ cells were isolated from human lung cancer A549 and H460 cells by Macs. Before sorting, the positive rate of CD133 are 2.8% and 2.4%. And after sorting, the positive rate of CD133 are 96.4% and 97.8% in A549 cells and H460 cells, respectively. **e**, **f** The tumor sphere volume of A549/CD133^+^ and H460/CD133^+^ cells were inhibited by SFN in a dose-dependent manner

To explore the role of SFN in lung cancer cells, we first detected cell viability through the MTT assay. The result showed that SFN could inhibit the growth of lung cancer A549 and H460 cells, which was clearly indicated by a declined cell viability trend in a dose-dependent manner following treatment with different concentrations of SFN (Fig. 1a). Electron microscope also shows a significant decrease in the density of A549 and H460 cells following treatments with 8 μmol/L SFN for 48 h (Fig. 1b).

To investigate whether SFN were sensitized to lung CSCs, we first isolated CD133^+^ cells from the human lung cancer A549 and H460 cells by MACS. CD133^+^ cells accounted for 96.4% and 97.8% in A549 and H460 cells after magnetic sorting (Fig. 1c, d). The CD133^+^ cells can form a colony in the tumorsphere culture medium. In sphere formation assay, the A549/CD133^+^ and H460/CD133^+^ cells were treated with 0, 4, 8 and 12 μmol/L SFN. At the end of 14 days, the volume of tumorspheres were measured, and the volume of tumorsphere trend showed a gradual decrease with increasing dose of SFN (Fig. 1e, f). These results showed that SFN can effectively inhibit self-renewal of lung LSCs.

### High expression of SHH signaling pathway and PHC3 in lung CSCs

**Fig. 2:**
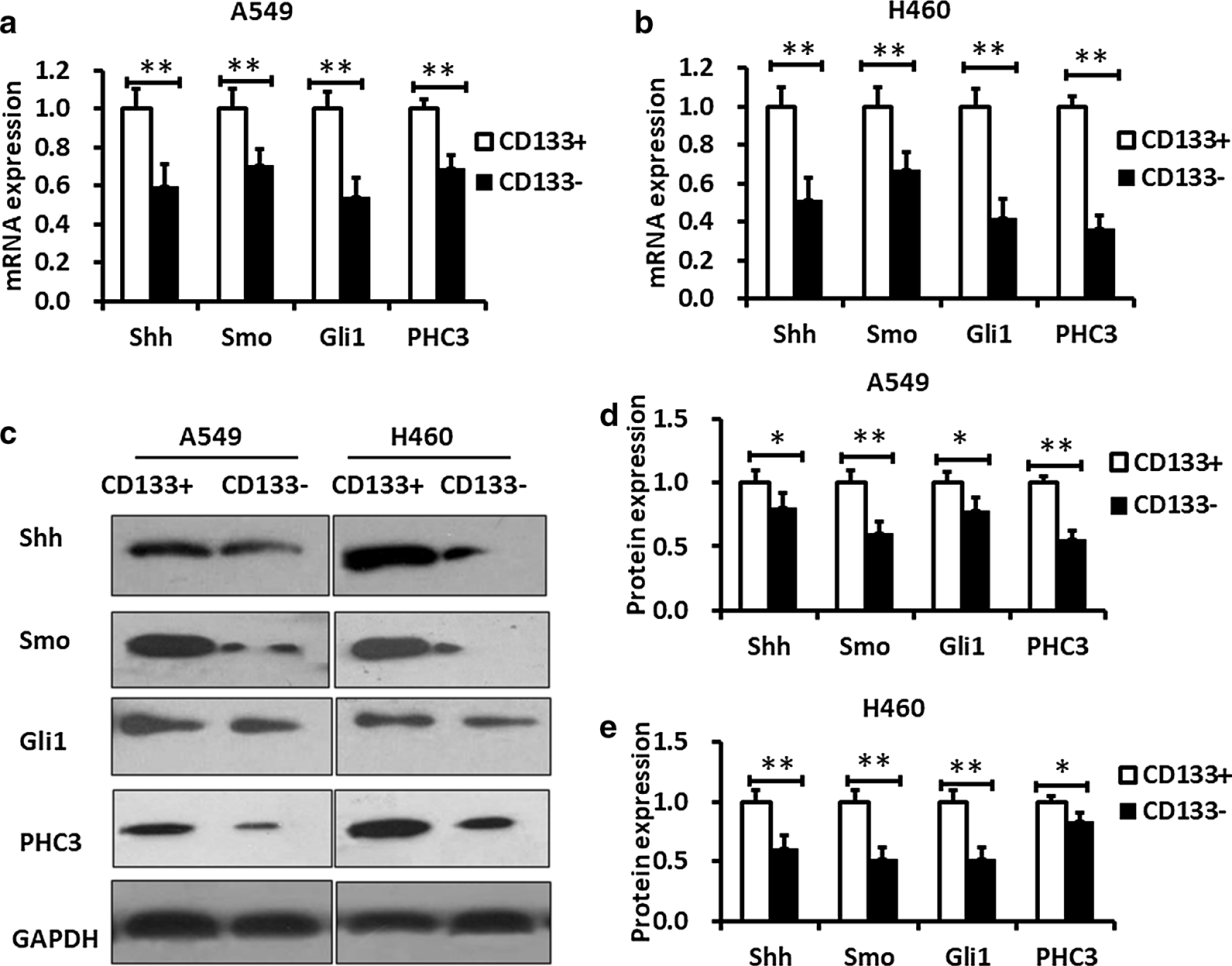
Expression of Shh, Gli1, Smo and PHC3 in CD133^+^ and in CD133^−^ lung cancer cells. **a**, **b** QPCR data shows that the mRNA levels of Shh, Gli1, Smo and PHC3 in A549/CD133^+^ and H460/CD133^+^ are higher than those in A549/CD133^−^ and H460/CD133^−^. **c**–**e** Western blotting data shows that the protein levels of Shh, Gli1, Smo and PHC3 in A549/CD133^+^ and H460/CD133^+^ cells are higher than those in A549/CD133^−^ and H460/CD133^−^ cells. The relative protein levels were quantified with Image J software. N = 3, Data are presented as mean ± SD, *P < 0.05, **P < 0.01, ***P < 0.001

It has been reported that the SHH signaling pathways and Polycomb group molecule PHC3 play an important role in the control of stem cell growth (Clarke et al. 2008; Subramani et al. 2017). However, the role of the SHH signaling pathways and PHC3 in lung CSCs is uncertain. The mRNA level of the important components of the SHH signaling pathway such as Shh, Smo and Gli1 was markedly increased in human lung CSCs (A549/CD133^+^ and H460/CD133^+^ cells) compared to that in human non-lung stem cells (A549/CD133^−^ and H460/CD133^−^ cells) (*P* < 0.01) (Fig. 2a, b). We also measured PHC3 expression level in CD133^+^ cells and CD133^−^ cells. PHC3 mRNA expression level was also markedly increased in CD133^+^ cells compare to CD133^−^ cells (*P* < 0.01).

To further determine whether the SHH signaling pathways and PHC3 had an abnormal expression in human lung CSCs, we examined the protein expression level of Shh, Smo, Gli1 and PHC3 in CD133^+^ cells and in CD133^−^ cells. Interestingly, Shh, Smo, Gli1 and PHC3 protein expression levels were consistent with mRNA transcription level (Fig. 2c, d, e). These data indicated that Shh signaling pathway and Polycomb group molecule PHC3 were abnormally activated in lung cancer stem cells.

### SFN regulates SHH signaling pathway and PHC3 in lung CSCs

**Fig. 3:**
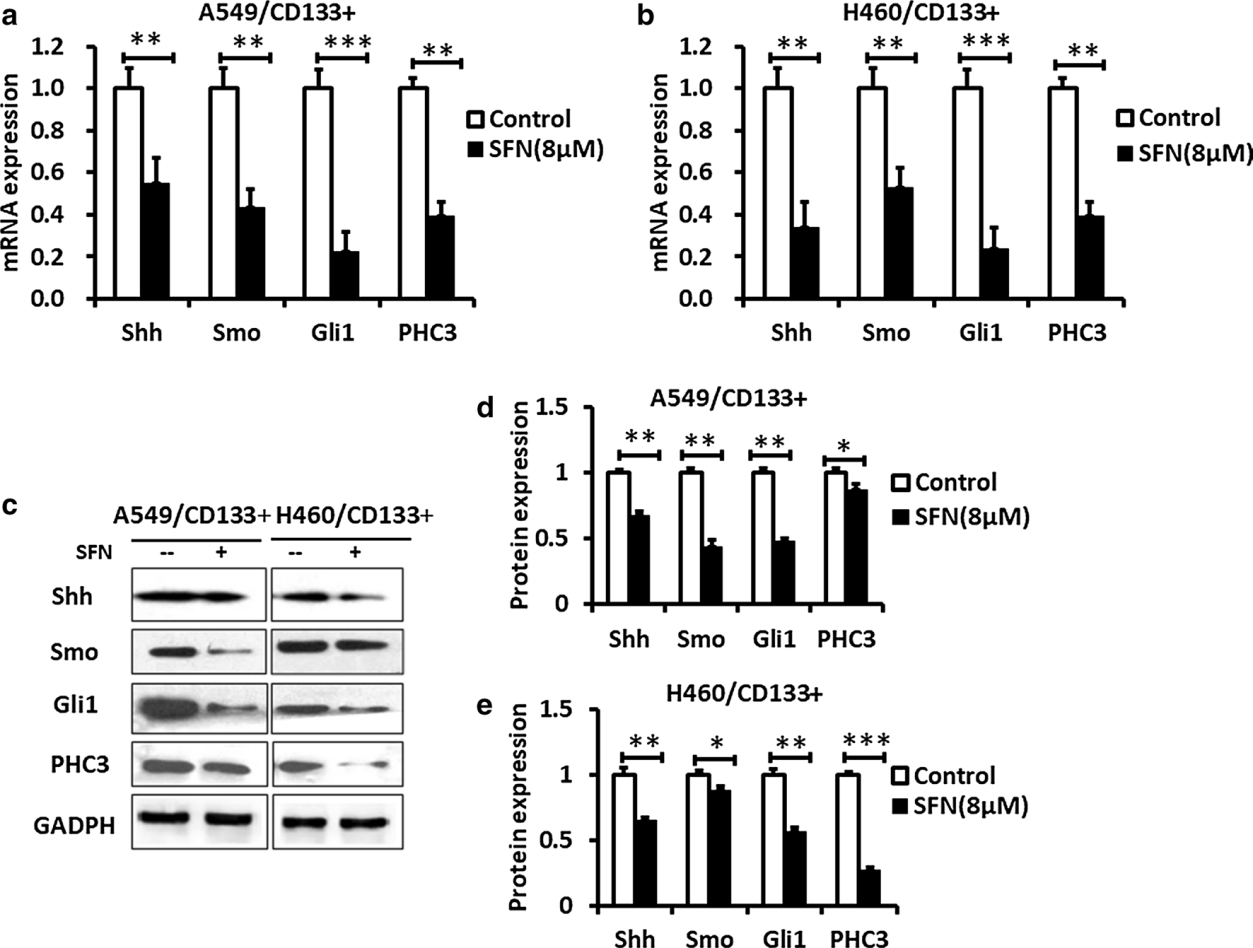
SFN inhibits expression of Shh, Gli1, Smo and PHC3 in human lung CSCs. **a**, **b** QPCR data shows that the mRNA levels of Shh, Gli1, SMO and PHC3 were significantly inhibited when lung CSCs were treated with SFN, compared with the control group. **c**–**e** Western blotting data shows that the protein levels of Shh, Gli1, SMO and PHC3 were significantly inhibited when lung CSCs were treated with SFN, compared with the control group. N = 3, Data are presented as mean ± SD, *P < 0.05, **P < 0.01, ***P < 0.001

To further explore whether SFN regulated SHH signaling pathway and PHC3 in lung CSCs, we examined the effects of SFN on the mRNA expression level of Shh, Smo, Gli1 and PHC3 in CD133^+^ lung cancer cells. As shown in Fig. 3a,b, SFN could markedly inhibit the mRNA expression of Shh, Smo, Gli1 and PHC3 in CD133^+^ cells (*P* < 0.01 or *P* < 0.001). The expression of these components was also further confirmed by western blotting assay. SFN could also inhibit protein expression of Shh, Smo, Gli1 and PHC3 in the lung cancer stem cells (*P* < 0.01or *P* < 0.001), (Fig. 3c–e). These data suggested that SFN can regulate by SHH signaling pathway and PHC3 in lung CSCs.

### SFN regulates self-renewal of lung CSCs through SHH signaling pathway and PHC3

**Fig. 4:**
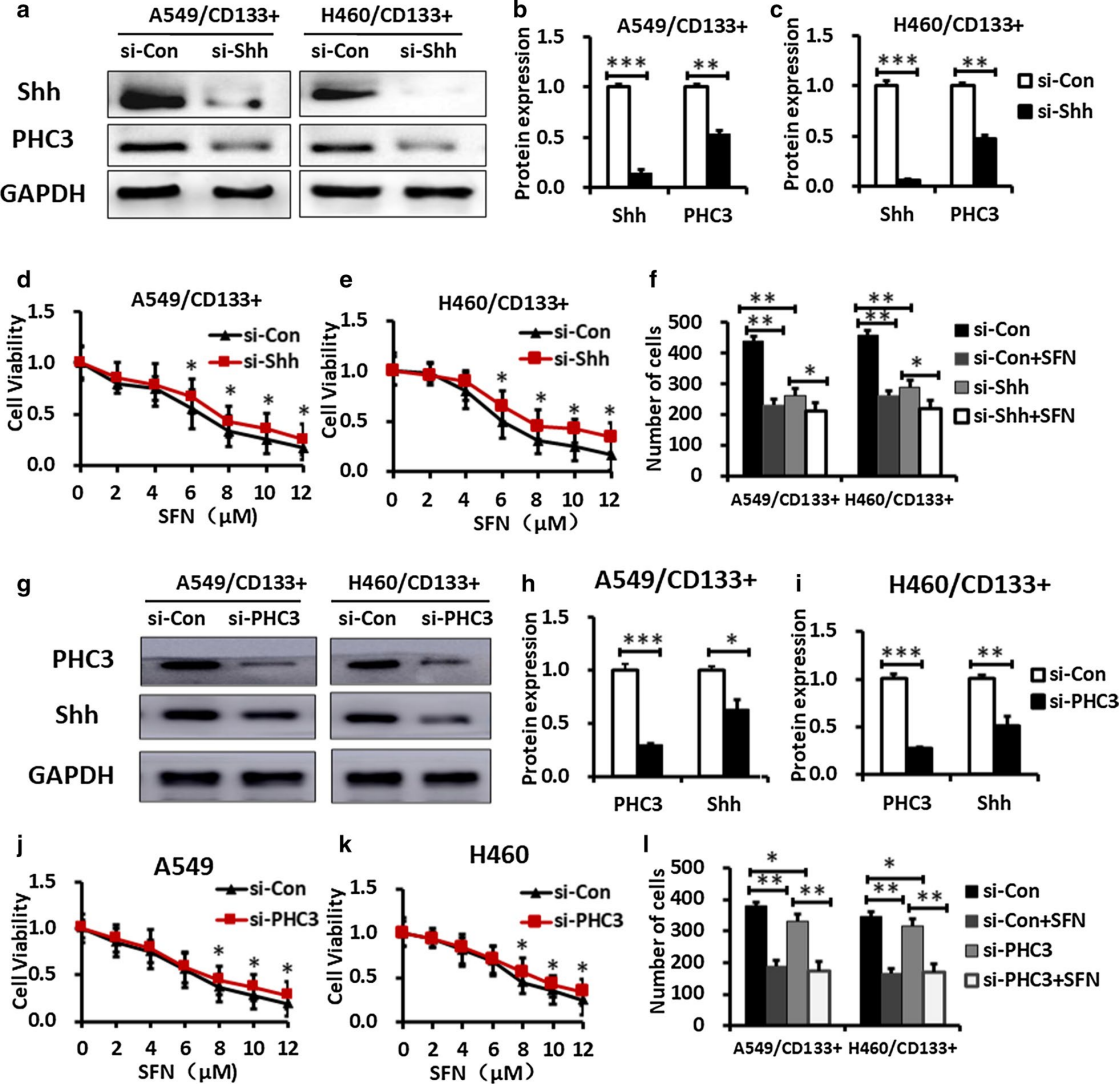
SFN regulates self-renewal of lung CSCs through SHH Signaling Pathway and PHC3. **a**–**c** The efficiency of siRNA to silence Shh gene were determined by Western blotting. The relative protein levels were quantified with Image J software. **d**, **e** SFN inhibited cell viability of siRNA-Shh group less than that of si-control group when SFN concentration was 6, 8, 10 and 12 μmol/L. **f** Cell numbers of siRNA-Shh cells are significantly less than that of si-control group. And the inhibitory effect of SFN on siRNA-Shh cells are significantly lower than that of si-control group. **g**–**i** The efficiency of siRNA to silence PHC3 gene were determined by Western blotting. The relative protein levels were quantified with Image J software. **j**, **k** SFN inhibited cell viability of siRNA-PHC3 group less than that of si-control group when concentration was 8, 10 and 12 μmol/L. **l** Cell numbers of siRNA-PHC3 cells are significantly less than that of si-control group. And the inhibitory effect of SFN on siRNA-PHC3 cells are significantly lower than that of si-control group. N = 3, Data are presented as mean ± SD, *P < 0.05, **P < 0.01, ***P < 0.001

In order to further identified the role of the SHH signaling pathway in self-renewal of lung CSCs, we silenced both Shh gene and PHC3 gene in A549/CD133^+^ and H460/CD133^+^ cells. Cells were transfected with siRNAs and maintained for 48 h. The transfection efficiency was determined by western blotting. The Shh protein expression level in siRNA-Shh transfected cells was suppressed compared with the control (Fig. 4a–c). And the PHC3 protein expression level in siRNA-PHC3 transfected cells was also suppressed compared with the control (Fig. 4g–i).

When the Shh gene was silenced successfully, the cell viability and number of cells in siRNA-Shh group were lower than those in si-control group (Fig. 4f). Further research revealed that the inhibitory effect of SFN on cell proliferation still exists in siRNA-Shh cells. The cell viability also showed a gradual decrease with increasing dose of SFN both in siRNA-Shh group and si-control group. SFN inhibited cell viability of siRNA-Shh group less than that of si-control group when the concentration were 6, 8, 10 and 12 μmol/L (P < 0.05) (Fig. 4d, e). At the same time, the number of cells decreased in both siRNA-Shh group and si-control group with SFN treatment, but the decrease in siRNA-Shh group was weaker than that in si-control group (Fig. 4f). These results indicate that downregulation of Shh attenuates the inhibitory effect of SFN on self-renewal of lung CSCs.

As no prior report on the relationship between SHH and PHC3 in lung cancer existed, we employed a further study expression of PHC3 in siRNA-Shh transfected cells. Interestingly, PHC3 expression level showed a significant decrease in siRNA-Shh compared with si-control in A549/CD133^+^ (*P* < 0.01) and H460/CD133^+^ cells (*P* < 0.01) (Fig. 4a–c). At the same time, the expression of Shh protein level in siRNA- PHC3 cells was also lower than that in si-control cells in A549/CD133^+^ (*P* < 0.05) and H460/CD133^+^ cells (*P* < 0.01) (Fig. 4g–i). And the proliferation and self-renewal ability of cells was reduced when PHC3 was silenced in A549/CD133^+^ (*P* < 0.05) and H460/CD133^+^ cells (*P* < 0.05). And the effect of SFN on the proliferation and self-renewal of siRNA-PHC3 cells was also lower than that of si-control cells. As shown in Fig. 4j, k, SFN inhibited cell viability of siRNA-PHC3 group less than that of si-control group when the concentration were 8, 10 and 12 μmol/L (*P* < 0.05). Cell numbers of siRNA-PHC3 cells are significantly less than that of si-control group following 48 h transfection. And the inhibitory effect of SFN on siRNA-PHC3 cells are significantly lower than that of control group (Fig. 4l). These data suggested that there was a cross link between SHH signaling pathway and PHC3, and both of which were involve in self-renewal of lung CSCs.

## Discussion

A series of scientific studies have shown that SFN can induce apoptosis in a variety of human cancers (Mi et al. 2009), leading to cell cycle arrest and inhibition of malignant growth. SFN is an isothiocyanate extracted from cruciferous vegetables. In this study, we observed that SFN could inhibit the activity of lung cancer A549 and H460 cells.

CD133 is an important marker of CSCs, which has the important characteristics of lung CSCs (Pavon et al. 2018; Kim et al. 2019). Therefore, we separated lung CSCs which express CD133^+^ from the human lung cancer A549 and H460 cells. To investigate whether SFN affected lung CSCs, we performed the sphere formation assay. We observed that the volume of tumorspheres were gradually decreased with the increasing dose of SFN. This result indicates that SFN has the potential to inhibit self-renewal of lung CSCs. However, the mechanism of the phenomenon still needs to be elucidated.

In recent years, several studies have reported that the SHH signaling pathway plays a critical role in the development and progression of many kinds of malignant tumours, such as gastric cancer (Yang et al. 2018) and lung cancer (Giroux et al. 2018; Maitah et al. 2011). It has also been reported that the SHH signaling pathway could regulate self-renewal and proliferation of CSCs and enhance tumor invasiveness (Zhu et al. 2017). In this study, we have demonstrated that the SHH signaling pathway was abnormally activated in lung CSCs, suggesting that the hyperactive SHH signaling may regulate the expression of stemness genes in lung cancer and play an important role in self-renewal and progression of lung CSCs.

To explore whether SFN inhibited self-renewal of lung CSCs through the SHH signaling pathway, we further investigated the expression of key components of the SHH signaling pathway in CD133^+^ lung cancer cells which were treated by SFN. The results showed that SFN can obviously reduce the mRNA and protein expression of Shh, Smo and Gli1 in CD133^+^ cells. Furthermore, when the Shh gene was silenced successfully in CD133^+^ lung cancer cells, the proliferation ability of siRNA-Shh cells was decreased as compared to that of the si-control group. And cell viability assay showed that the inhibitory effect of SFN on cell proliferation in the siRNA-Shh cells was much weaker than that in the si-control cells. This work demonstrated that the actions of SFN to inhibit self-renewal of lung CSCs is achieved by regulating SHH signaling pathway.

PHC3 is one of the members of PRC1, is essential in early embryonic development and has been implicated in embryonic stem cell pluripotency (Pachano et al. 2019; Kloet et al. 2016). It has been reported that PHC1 was important in regulating stem cells (Hou et al. 2019; Linxweiler et al. 2012). Other previous studies have shown that PcG complexes control cellular proliferation and favor tumorigenesis (Chan and Morey 2019; Wang et al. 2015). AM Iwata et al. (2010) reported that PHC3 expression was abnormal in osteosarcoma. In this study, we investigated whether PHC3 like PHC1 plays the same role in regulating lung cancer stem cells. Our data indicated that both the mRNA and protein expressions of PHC3 were also markedly increased in human lung CSCs. Further research also showed that SFN inhibited the mRNA and protein expressions of PHC3 in CD133^+^ cells. Moreover, PHC3 presented the same expression pattern as SHH signaling pathway in lung CSCs. However, there was no evidence to support the interaction between SHH and PHC3. Therefore, we investigated the protein expression of PHC3 in Shh silenced cells. Here, we demonstrated that downregulation of Shh suppressed PHC3 protein expression in A549 /CD133^+^ and H460/CD133^+^ cells. At the same time, the expression of Shh protein level in PHC3 silenced cells was also lower than that in control cells. And the proliferation ability was reduced when PHC3 was silenced in A549/CD133^+^ and H460/CD133^+^ cells. Remarkably, both gene silencing of Shh and PHC3 have been implicated in the cell proliferation process. Above all, these data confirmed that there was a cross-talk between SHH signaling pathway and PHC3, and both SHH signaling pathway and PHC3 were involve in self-renewal of lung CSCs. Collectively, these data indicate that SHH signaling pathway and PHC3 play important roles in regulatory effect of SFN in self-renewal of lung CSCs.

In conclusion, this study provided evidence that SFN could serve as a potent anticancer agent and inhibit proliferation of lung cancer and self-renewal of lung CSCs. Furthermore, this study revealed that SHH signaling pathway and PHC3 worked together in lung CSCs and that aberrant activation of these signals promoted the tumorigenesis and progression of lung cancer. These findings suggested that SFN could be exploited in lung cancer treatment by means of regulating the SHH signaling pathways and PHC3.

## Data Availability

The datasets used and/or analyzed during this study are available from the corresponding author on reasonable request.

## Abbreviations

SFN: Sulforaphane
CSCs: Cancer stem cells
Shh: Sonic Hedgehog
Smo: Smoothened
PHC3: Human polyhomeotic homolog 3
PcG: Polycomb group
FACS: Flow cytometry assays
MTT: 3-(4,5)-Dimethylthiahiazo (-z-y1)-3,5-di- phenytetrazoliumromide
QPCR: Quantitative reverse transcription polymerase chain reaction
PcG: Polycomb group
SDS-PAGE: Sodium dodecyl sulfate polyacrylamide gel electrophoresis
PVDF: Polyvinylidene fluoride
ECL: Efficient chemiluminescence kit
PRC: Polycomb repressive complex 1
